# Exploring plasmonic gradient metasurfaces for enhanced optical sensing in the visible spectrum

**DOI:** 10.1515/nanoph-2023-0809

**Published:** 2024-01-15

**Authors:** Shih-Hsiu Huang, Pin Chieh Wu

**Affiliations:** Department of Photonics, National Cheng Kung University, Tainan 70101, Taiwan; Center for Quantum Frontiers of Research & Technology (QFort), National Cheng Kung University, Tainan 70101, Taiwan; Meta-nanoPhotonics Center, National Cheng Kung University, Tainan 70101, Taiwan

**Keywords:** enhanced optical sensing, plasmonic gradient metasurface, scattered light intensity ratio, sensitivity

## Abstract

While conventional optical sensors hold historical significance, they face inherent limitations in sensitivity, operational intricacies, and bulky size. A breakthrough in this realm comes from the advent of metasurface sensors, which leverage nanoscale optical effects, thereby expanding the horizons of optical sensing applications. However, past methods employed in metasurface sensors predominantly rely on wavelength shifts or intensity changes with high-*Q* resonances, thereby significantly restricting the detection bandwidth. In response to these challenges, this study introduces a plasmonic gradient metasurface-based sensor (PGMS) designed for refractive index detection across a wide wavelength spectrum. Through the utilization of the Pancharatnam–Berry phase method, the PGMS achieves a distinctive 2*π* phase shift, facilitating the simultaneous generation of specular and deflected beams. The introduction of a far-field intensity ratio (I* = *I*_+1_/*I*_0_) amplifies the change in optical response by maximizing the deflected beam’s intensity while minimizing specular reflection. Experimental validation attests to the PGMS’s consistent performance across diverse media and wavelengths, successfully overcoming challenges associated with oxidation issues. Furthermore, the incorporation of a normalization factor enhances the PGMS’s sensing performance and versatility for broadband optical sensing, accommodating variations in the refractive index. Particularly sensitive in green wavelengths, the PGMS demonstrates its potential in visible spectrum applications, such as biomedical diagnostics and environmental monitoring. This research not only addresses challenges posed by conventional sensors but also propels optical sensing technologies into a realm of heightened sensitivity and adaptability.

## 1 Introduction

Traditional optical sensors have historically played a crucial role in the fields of science and engineering, offering precise measurement capabilities and widespread applicability. However, they do come with inherent limitations, including sensitivity constraints to minute variations and challenges when deployed in complex environments. Moreover, they often necessitate bulky equipment or intricate optical setups, resulting in increased costs and operational complexity. In response to these limitations, metasurface sensors have emerged. These sensors leverage nanoscale optical effects by fine-tuning the design of meta-atoms, granting them exceptional flexibility and tunability [1]–[3]. Consequently, metasurface sensors represent a significant technological breakthrough, expanding the application horizons of optical sensing.

Metasurface sensors possess substantial application value and significance across a multitude of fields today. They harness nanophotonic resonances and phenomena within metals and high-index semiconductors, merging the realms of optics and electronics to achieve exceptional sensitivity in spectral reconstruction [4], [5], polarization detection [6], [7], depth and edge characterization [8]–[10], and molecular fingerprint retrieval [11]. Their impact extends to contemporary domains such as science, medicine, environmental monitoring, and information technology. Notably, the application of metasurface sensors in refractive index detection [12], [13] and the biomedical sector [11], [14], [15] holds particular significance. These sensors enable the detection of minute concentrations of biomolecules [16], offering substantial potential for early drug screening and advancements in biomedical research [17]. Moreover, metasurface sensors provide real-time [18], [19], label-free sample analysis [20], supplying vital tools for clinical diagnostics and epidemic surveillance [21].

Within the domain of metasurface sensors for refractive index detection, two fundamental scenarios are in play. The first scenario centers on the resonant wavelength shift when nanostructures are placed in different media [22]–[24]. To observe these shifts in resonant wavelengths clearly, it is crucial for the designed nanostructures to have a relatively high quality factor (*Q* factor) in the optical spectra [25]–[27]. Indeed, a high *Q* factor not only aids in this observation but also enhances the figure of merit (FOM) [28]–[30], subsequently improving sensing performance. The development and optimization of resonant meta-atoms, including specific resonant modes like Mie resonances [31]–[33], toroidal modes [34]–[36], and non-radiative anapole modes [37], [38], have enabled strong near-field enhancement and confinement. This innovative approach has the potential to significantly enhance sensitivity when detecting changes in refractive index. Notably, these resonances often exhibit a relatively high *Q* factor, further contributing to their effectiveness in sensing applications. However, it is important to note that refractive index detection based on wavelength shifts can introduce measurement errors, especially when the medium to be detected exhibits high dispersion within the working bandwidth. The second scenario involves detecting changes in refractive index by characterizing optical intensity at a fixed wavelength [39]–[41]. Consequently, measurement accuracy is higher for dispersive media. Nevertheless, to enhance the intensity difference for different media, the resonant feature must, once again, possess a relatively high *Q* factor [42]. The requirement for a high *Q* factor is less desirable when the goal is to expand the working bandwidth of metasurface sensors, which applies to both of the abovementioned approaches.

Previous research has delved into the development of broadband metasurface sensors employing high *Q* factor nanostructures. For instance, in order to discern the unique signatures of biomolecules across a wide range of wavelengths, scientists have employed a strategy that involves interleaving metasurface chips [14], [16], [43]. Each individual chip is designed to perform sensing at a specific wavelength. Nonetheless, the approach of using interleaved metasurfaces has the potential to increase the device’s footprint, thereby sacrificing one of the key advantages of compact dimensions associated with metasurfaces. While designing metasurfaces with multi-resonant features [44] or angle-dependent response [11] is a practical approach for broadband sensing, the specific requirements for spectral response can complicate the optimization process of the metasurface [45]. Thus, the challenge of striking a balance between sensitivity enhancements and working bandwidth remains an ongoing concern that researchers are actively addressing as they strive to achieve the optimal performance of metasurface sensors.

**Figure 1:**
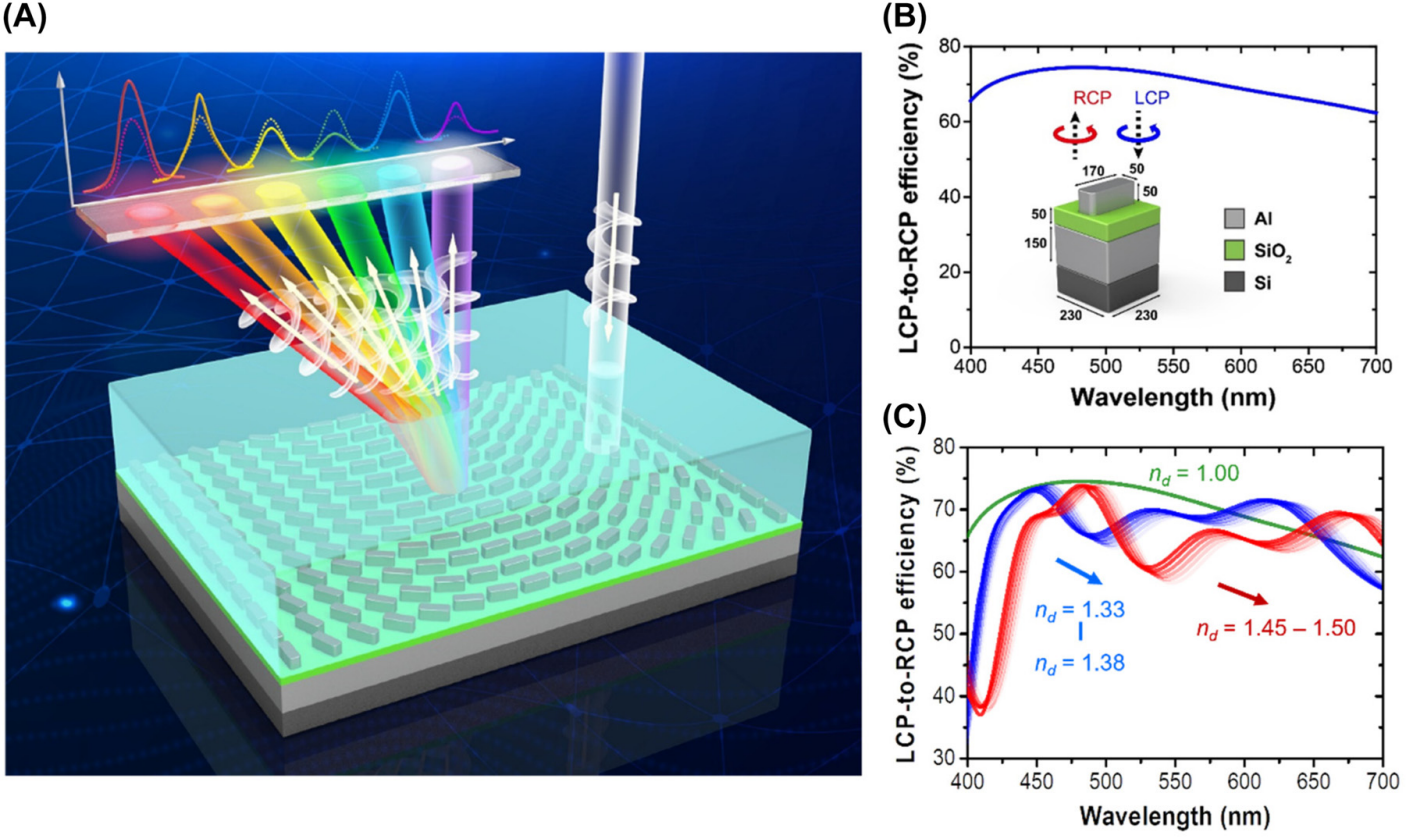
Design for the Al-based meta-atom of the PGMS. (A) A schematic illustration demonstrates the refractive index sensing mechanism utilizing a plasmonic gradient metasurface-based sensor. Variations in the environmental refractive index are observed to alter the intensity of the deflected beam (*I*_+1_), thereby offering a pathway for material characterization. This sensor allows the detection of optical responses at various wavelengths within the same image plane. (B) The simulated LCP-to-RCP conversion efficiency of the Al meta-atom. Inset: the geometric dimensions of designed meta-atom (unit: nm). (C) Simulated circular-polarized conversion efficiency of the meta-atom within its surrounding environment is examined across a range of refractive indices *n*_d_.

In this study, we propose and experimentally present a plasmonic gradient metasurface-based sensing platform capable of refractive index detection across a wide spectrum of wavelengths (refer to Figure 1A for the conceptual schematic). The incorporation of a phase gradient facilitates the generation of both specular and deflected beams in the far field, providing multiple parameters for signal detection and analysis. To achieve a 2*π* phase modulation across a wide wavelength spectrum, we employ the Pancharatnam–Berry (PB) phase method in the construction of the gradient metasurface. In our quest to bolster detection sensitivity, we introduce a figure of merit defined as the far-field intensity ratio I* of the specular and deflected beams: I* = *I*_+1_/*I*_0_. This approach amplifies the change in optical response by maximizing the intensity of the deflected beam (*I*_+1_) while minimizing the specular reflection intensity (*I*_0_). In conjunction with the plasmonic resonant characteristics and phase gradient response, this method ensures high sensitivity throughout the visible wavelength range, as assessed by evaluating the normalized intensity ratio $\left(I_{\text{nor}}^{*}\right)$ . Furthermore, the inherent property of the gradient metasurface ensures that the angle of the deflected beam remains constant when different analytes are in close proximity to the metasurface. This feature proves highly advantageous for sensing applications as it allows for a fixed detector position, simplifying the detection process without the need of any spectrometer or costly instruments. This plasmonic gradient metasurface-based sensor (PGMS) holds significant promise for a wide range of applications, including biomedical diagnostics and environmental monitoring, and it paves the way for advanced and highly sensitive optical sensing technologies in the future.

## 2 Results and discussion

Figure 1B shows the simulated circular cross-polarized reflection spectrum of the proposed plasmonic meta-atom for the PGMS, with the inset highlighting the optimized physical dimensions of the meta-atom. Notably, the plasmonic meta-atom exhibits a polarization conversion efficiency exceeding 60 % across the spectral range from 400 to 700 nm. Before delving into the practicality of optical sensing using gradient metasurfaces, our initial focus is on investigating the optical response of the plasmonic meta-atom in various surrounding environments. It is important to emphasize that while achieving broadband high conversion efficiency is advantageous for high-performance metasurface components and devices, it is less suitable for sensing applications across a wide spectrum. As shown in Figure 1C, the characteristics of the circular cross-polarized spectrum undergo a significant transformation, shifting from a relatively broad high reflection to multiple peaks and dips as the environmental refractive index varies from 1 to 1.33. These variations result from the diverse strengths of magnetic resonances, which, in turn, are a consequence of the dipole-dipole coupling between the topmost meta-atom and the back reflector (see Supporting Information for field profiles). This striking variation presents a challenge when attempting to distinguish resonant peak shifts, especially for those who prefer to rely on wavelength shifts as the figure of merit for sensing. Although there is a substantial intensity difference at certain wavelengths, the reflection intensity remains almost constant at multiple spectral positions. This minimal intensity variation complicates the task of achieving broadband optical sensing. When the changes in the environmental refractive index are relatively small, such as within the range of 1.33–1.38 or from 1.45 to 1.5, the spectral features remain relatively stable. Nevertheless, a slight change in the resonant wavelength shift (or intensity change at a fixed wavelength) is still noticeable at several spectral positions. These findings highlight that highly-reflective broadband meta-atoms may not be the most suitable option for creating effective broadband metasurface sensors.

To enhance the optical sensing performance across the entire visible spectrum, we have proposed the utilization of a plasmonic gradient metasurface as the sensing platform. In this study, the PB phase method is employed to achieve a 2*π* phase shift within a supercell, and this choice is motivated by two key reasons. Firstly, the PB phase offers a highly promising solution due to its dispersionless property, making it effective in generating beam deflection across a wide frequency range. Secondly, by relying on the structural orientation angle for realizing the phase shift with the PB phase method, we can maintain the high reflection characteristics of the designed meta-atom when using it to construct the gradient metasurface. Based on PB phase theory, the 2*θ* phase shift of the cross circularly-polarized component can be attained by rotating the nanostructure with an orientation angle *θ*. In fact, the phase shift linked to the orientation angle can be expressed in the circular basis using the Jones matrix as:

(1) $$\left[\begin{array}{c} E_{\text{rLCP}}\\ E_{\text{rRCP}} \end{array}\right]=\left[\begin{array}{cc} J_{\text{LL}} & J_{\text{LR}}{\text{e}}^{\text{i}2\theta }\\ J_{\text{RL}}{\text{e}}^{-\text{i}2\theta } & J_{\text{RR}} \end{array}\right]\left[\begin{array}{c} E_{\text{iLCP}}\\ E_{\text{iRCP}} \end{array}\right]$$

where *J*_LL_, *J*_LR_, *J*_RL,_ and *J*_RR_ represent the conversion coefficient of different components. *E*_r_ and *E*_i_ represent the reflected electric field and incident electric field, respectively. LCP and RCP is denoted for left-handed circular polarization and right-handed circular polarization, respectively. More details and discussions can be found in Supplementary Note 1.

**Figure 2:**
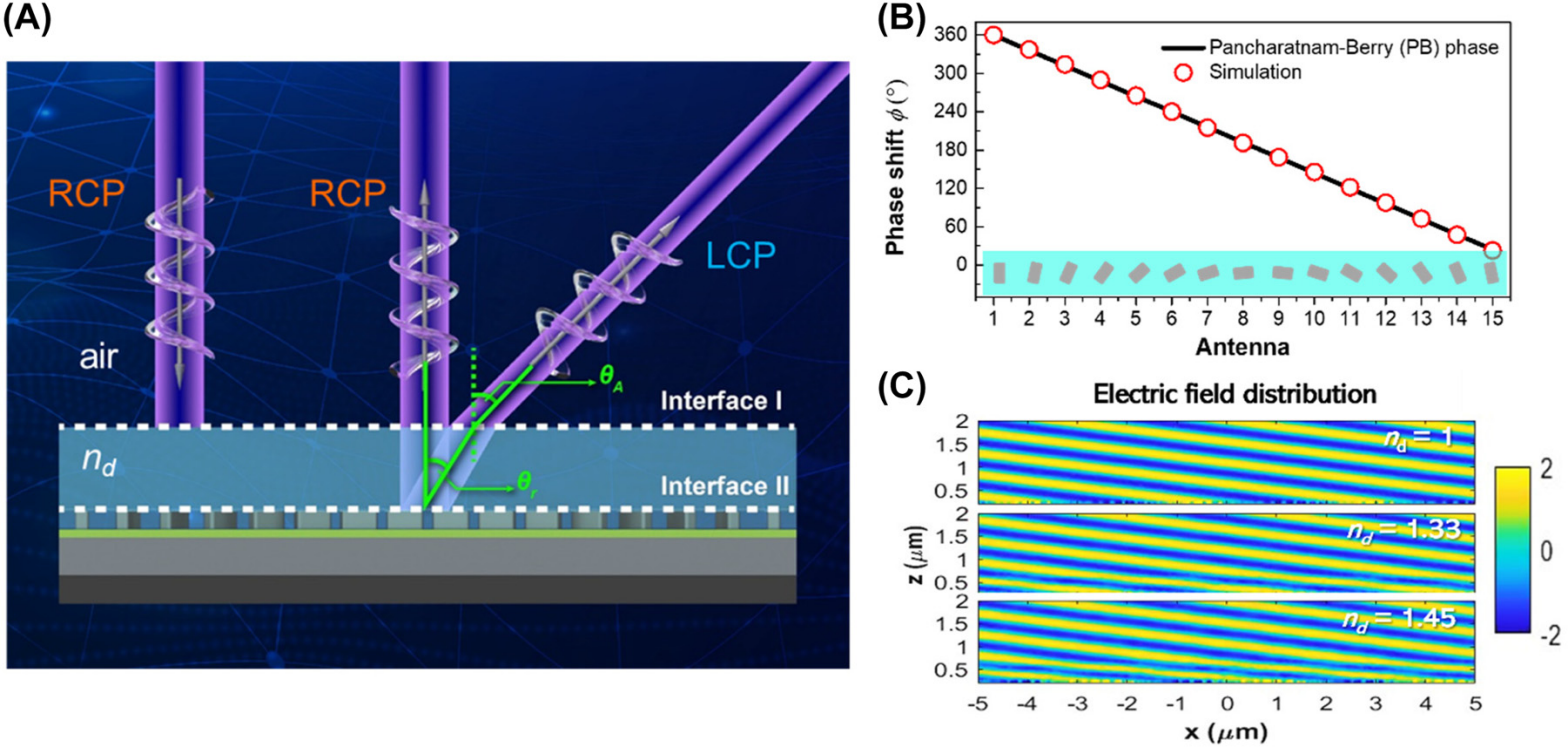
Working mechanism of the PGMS. (A) The schematic illustration of refractive index sensing within an analyzed medium (light blue region) with refractive index represented as *n*_d_. When RCP light is incident, it results in the simultaneous generation of spectral reflection with RCP and a deflected beam with LCP light. (B) Simulated phase shift across one supercell of the plasmonic gradient metasurface. (C) Simulated electric-field distribution (LCP component) of the PGMS within a medium with varying refractive indices. Incident wavelength: 532 nm.

When incident light carries RCP and interacts with the PB phase-based gradient metasurface, two distinct beams are generated in the far field: the co-polarized specular reflection and cross-polarized deflection, as schematically illustrated in Figure 2A. Please note that an identical optical response can be achieved when the incident light is in LCP. The only variation lies in the deflected angle, which shifts to the opposite side relative to the normal vector of the substrate. As a result, the PGMS consistently operates effectively under the condition of circularly-polarized illumination. Despite the reflected and deflected beams exhibiting orthogonal polarization states, their simultaneous differentiation in a single-shot imaging acquisition is facilitated by the wide range of scattering angles. This eliminates the need for a polarizer or imaging device. Moreover, the concurrent generation of two beams in reflection offers additional degrees of freedom as indicators for sensing applications. In fact, the deflected angle *θ*_r_ for a gradient metasurface at interface *I* (refer to Figure 2A) can be described by [46]–[48]:

(2) $$\theta_{r}=\sin^{-1}\left(\frac{\lambda_{0}}{2\pi n_{\text{d}}}\times \frac{\text{d}\phi }{dx}\right)$$

where *λ*_0_ is the working wavelength in free space, *n*_d_ is the refractive index of the medium to be detected, and $\frac{\text{d}\phi }{dx}$ represents the phase gradient along the *x*-axis. According to Eq. (2), the deflected angle *θ*_r_ can vary even when the incident wavelength and phase gradient $\frac{\text{d}\phi }{dx}$ are fixed. This variability may pose challenges during the optical sensing process, particularly under conditions where the spatial positions between specular and deflected beams are too close. However, we have discovered that the deflected angle *θ*_A_ observed in the far field remains constant when the PGMS is covered by an analyte layer with an arbitrary refractive index *n*_d_ even as *θ*_r_ varies (see additional details and discussions in Supplementary Note 2). This feature is highly advantageous for broadband metasurface sensors since the signal detected in the far field consistently corresponds to the wavelength of interest at its designed deflected angle, regardless of changes in the surrounding environment. To verify this point, we construct a plasmonic gradient metasurface using 15 meta-atoms with an orientation angle step of 12° between neighboring meta-atoms (refer to Figure 2B). As can be seen in Figure 2C, the propagation direction of the deflected beam remains unchanged in different surrounding environments. This further confirms the effectiveness of the plasmonic gradient metasurface as an optical sensor.

**Figure 3:**
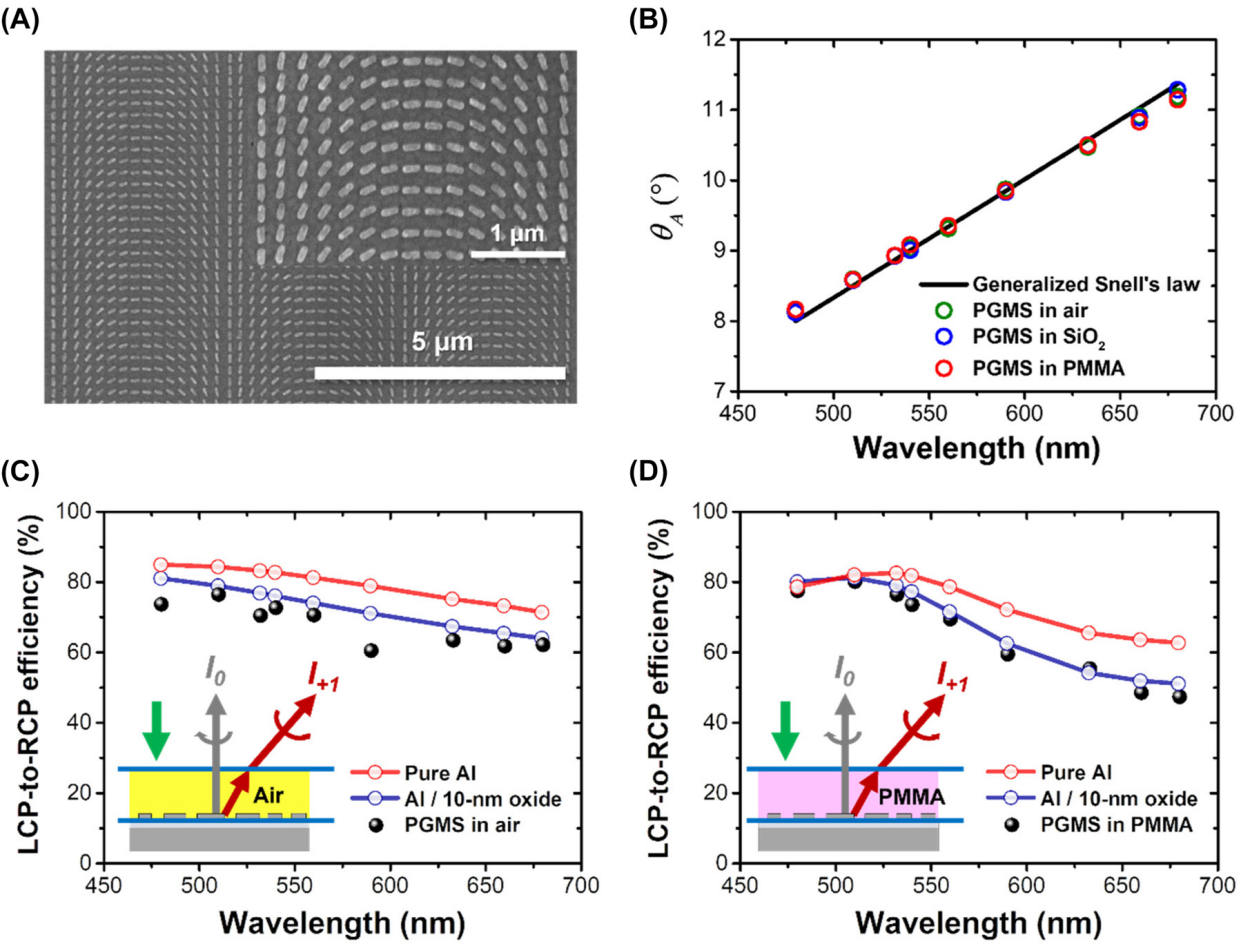
Experimental verification of the fabricated PGMS. (A) Top-view SEM images of the Al-based plasmonic gradient metasurface. (B) Angle of deflected beam as a function of the incident wavelength within the visible range as meta-sensor embedded in different surrounding environment. Color circles: measurement. (C, D) Simulated (red and blue circles) and measured (black dots) circular cross-polarization conversion efficiency as the PGMS embedded in (C) air and (D) PMMA.

**Figure 4:**
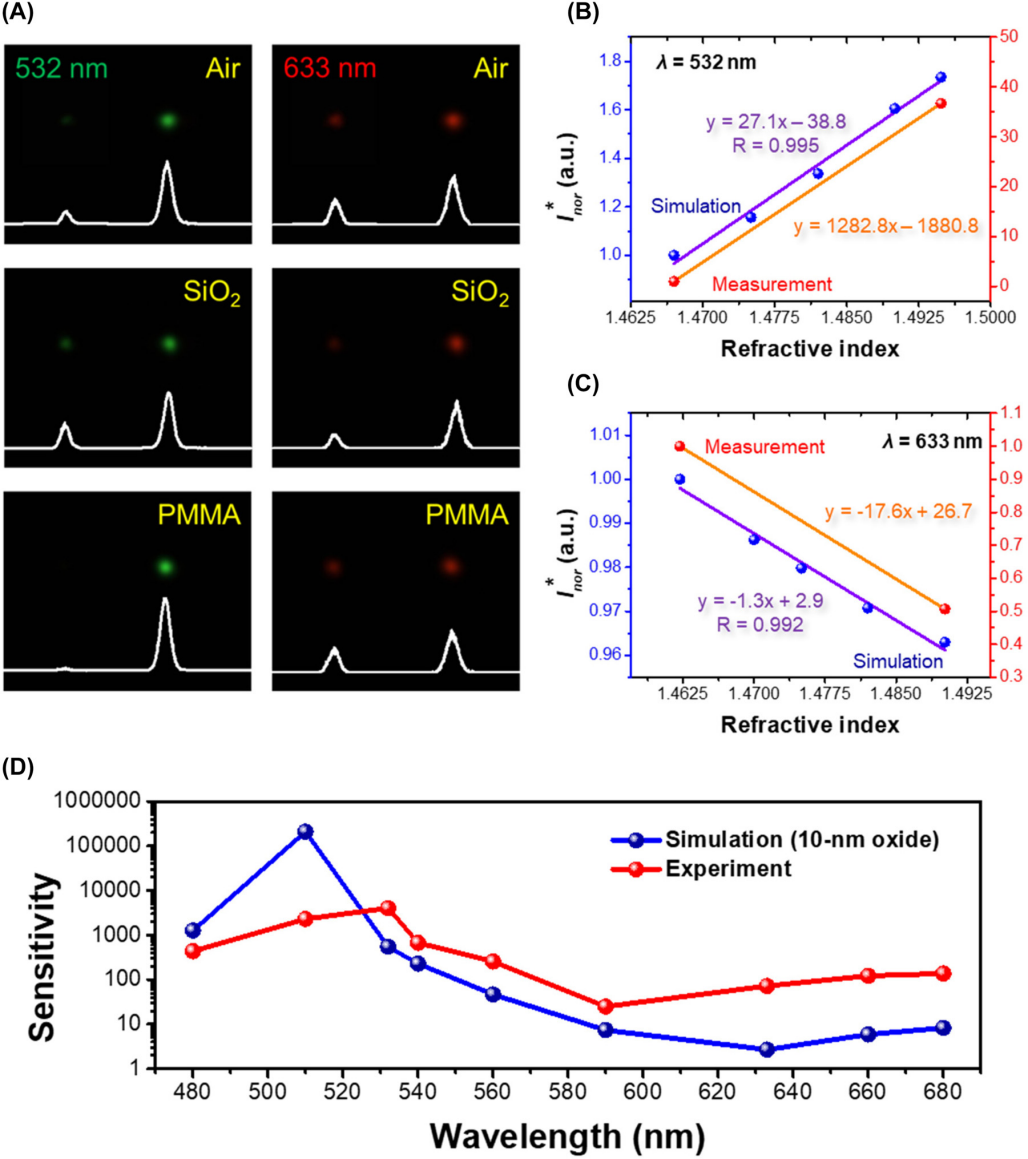
Sensing performance of the fabricated PGMS. (A) The far-field light intensity distributions at wavelengths of 532 nm and 633 nm for the PGMS when it is covered by different materials. (B, C) The simulated and measured normalized intensity ratios $I_{\text{nor}}^{*}$ in refractive index spanning from the values of PMMA to SiO_2_ at wavelengths of (B) 532 nm and (C) 633 nm. (D) The simulated and experimental sensitivity (logarithm scale) as a function of wavelength. A 10-nm-thick oxidation layer is applied to the surface of meta-atoms in the simulation.

Next, we fabricated an Al-based PGMS using a standard electron beam lithography process (refer to Section 4 for more details). We then implemented experimental characterization to assess its optical performance, specifically examining the deflected angle in different surrounding media and the efficiency of deflection. Due to the limitations of the acousto-optic tunable filter utilized in our study, we conducted experiments within the wavelength range of 480–680 nm. Figure 3A presents the scanning electron microscope (SEM) images of the fabricated PGMS, showing uniformity and precision in both physical shape and dimensions. The optical setup employed for characterization is illustrated in Figure S1. In Figure 3B, the measured deflected angle of the PGMS sample is plotted based on captured images at various wavelengths. Importantly, these measurements closely align with the theoretically predicted values, offering robust confirmation that the deflected angle remains remarkably consistent even when the PGMS is covered by different media, despite the occurrence of the oxidation issue with the Al nanostructures. This alignment serves to validate the earlier discussions and underscores the resilience of the PGMS in maintaining its optical performance under varying conditions. To experimentally characterize the beam deflection efficiency, we spatially integrated the intensity of incident beam spots within a fixed range by replacing the PGMS with a silver mirror in the optical setup (see Figure S1) as the reference. By evaluating the deflected beams through integrating the light intensity in captured images from Figures 4A and S8, the measured beam deflection efficiency can be calculated using the intensity ratio of deflected to incident beams. The comparison of simulated and measured optical deflection efficiency in free space is provided in Figure 3C. Clearly, there is a noticeable misalignment between the experimental values (black dots) and the simulated values using the bare Al nanostructure in simulations (red circles). This discrepancy is attributed to the oxidation issue in the fabricated Al-based PGMS. To address this, we implemented simulations considering a 10 nm-thick oxidation layer covering the meta-atoms. As depicted, the simulated deflection efficiency with this additional oxidation layer demonstrates closer values and a similar trend to the experimental results, supporting the earlier assumption. To delve deeper into the analysis, Figure 3D shows the measured and simulated beam deflection efficiency for the PGMS covered by a layer of PMMA photoresist. Similarly, the observed experimental trend closely mirrors the simulated results when considering a 10 nm-thick oxidation layer. Experimental and simulated outcomes for the PGMS covered by SiO_2_ are detailed in Figure S2. Figure S3 provides the refractive index values of PMMA photoresist and SiO_2_ used in simulations, which are measured using an ellipsometer. Despite a decline in beam deflection efficiency in real samples due to the oxidation issue, the sensitivity of the plasmonic gradient metasurface as an optical sensor remains relatively high. This is attributed to the spectral reflection intensity remaining low, as elaborated in the subsequent paragraph.

Lastly, we investigate how the surrounding environment affects the functionality of our PGMS platform. In Figure 4A, the measured scattered images at 532 nm and 633 nm are presented when the PGMS is covered by various media (refer to Figure S4 for additional results). Once again, it is evident that the angle of the deflected beam for the same wavelength remains constant even when the analyte layer is altered. Moreover, leveraging the physical characteristics of the gradient metasurface, the intensity of specular reflection *I*_0_ and deflected light *I*_+1_ can be simultaneously obtained without the need for any polarizer or spectrometer. This is achieved by spatially integrating the intensity of captured images within a fixed range depending on the working wavelength. One can see that *I*_0_ consistently registers a lower value than *I*_+1_ for all wavelengths, highlighting the high operational efficiency of the proposed plasmonic gradient metasurface for broadband beam deflection. Moreover, there is a discernible difference between both *I*_0_ and *I*_+1_ at the same wavelength when the surrounding environment undergoes changes. While this observation suggests that *I*_0_ and *I*_+1_ can serve as indicators for optical sensing, the relatively small fluctuations in scattered intensity impede the optimal sensing performance of the PSMG.

To enhance the sensing performance of the PSMG, we introduce a figure of merit defined by the scattered intensity ratio, denoted as I* = *I*_+1_/*I*_0_. The design of the PSMG aims to minimize *I*_0_ and maximize *I*_−1_, consequently substantially boosting the detected FOM. For a fair comparison, given the relatively smaller index of SiO_2_, we introduce the normalized intensity ratio $I_{\text{nor}}^{*}$ defined as:

(3) $$I_{\text{nor}}^{*}=\frac{I_{n_{\text{d}}}^{*}}{I_{\text{ref}}^{*}}$$

where $I_{n_{\text{d}}}^{*}$ and $I_{\text{ref}}^{*}$ represent the intensity ratio for the PGMS embedded in a material under examination and a reference medium, respectively. In this context, the reference medium is the SiO_2_. Figure 4B and C, respectively, plot the normalized intensity ratio $I_{\text{nor}}^{*}$ as a function of refractive index at wavelengths of 532 nm and 633 nm, respectively (see Figure S5 for more experimental results). With an increase in the refractive index of the surrounding environment, the optimized condition of the meta-atoms is disrupted. Consequently, the intensity ratio I* typically decreases when there are variations in the surrounding environment. However, the introduction of the normalization factor $I_{\text{ref}}^{*}$ disrupts this trend. As a result, $I_{\text{nor}}^{*}$ can either increase or decrease with an increase in the refractive index of the surrounding environment (refer to Figures 4B and C and S5). This significantly enhances the versatility of the PGMS for broadband optical sensing. Although it shows some discrepancy between simulated and experimental results, the trends of the results exhibit similar variation. For a more quantitative analysis of the sensing performance of the PGMS, we defined the sensitivity as:

(4) $$\text{Sensitivity}=\frac{\Delta I_{\text{nor}}^{*}}{\Delta n_{\text{d}}}$$

where ∆*n*_d_ represents the refractive index change. The numerical and experimental sensitivity as functions of incident wavelength are provided in Figure 4D. The similar spectral features observed in both measurement and simulation affirm the high sensing performance of the PGMS within the visible spectral range. The peak sensitivity is observed in the green wavelengths, can be attributed to the occurrence of the highest deflection efficiency within the same region. While the sensitivity values in the red region may appear relatively lower, they still consistently range between ∼10 and ∼700. These demonstrations affirm that the developed PGMS is exceptionally well-suited for broadband sensing in the visible spectrum.

## 3 Conclusions

In summary, our investigation introduces an innovative refractive index detection platform based on a plasmonic gradient metasurface that operates seamlessly across a broad range of wavelengths. Leveraging the PB phase method, our PGMS achieves a 2*π* phase shift within a supercell, paving the way for the generation of both specular and deflected beams in the far field. The PGMS emerges as a versatile and highly sensitive solution, with the introduction of the far-field intensity ratio I* (defined as *I*_+1_/*I*_0_) serving as a key performance metric. This metric strategically enhances the optical response by maximizing the intensity of the deflected beam *I*_+1_ while minimizing specular reflection *I*_0_. Experimental validations reinforce the robustness of the PGMS across various media and wavelengths, addressing challenges posed by oxidation issues in the Al nanostructures. The incorporation of a normalization factor further amplifies the PGMS’s adaptability for broadband optical sensing, enabling nuanced intensity variations with changes in the refractive index of the surrounding environment. The PGMS’s efficacy is underscored by its notable sensitivity, reaching peak performance in the green wavelengths. Even in regions with relatively diminished sensitivity values, spanning from ∼10 to ∼700 in the red spectrum, the PGMS consistently delivers reliable performance. While the smallest detected index change in this study is ∼0.028, we anticipate that the PGMS’s high sensitivity, particularly for green wavelengths, suggests the potential for detecting even smaller refractive index changes. Considering the dependence of our proposed metasurface on the gap plasmon configuration, a potential concern arises where the intensified plasmon field might leakage into the dielectric spacer, thereby diminishing sensitivity. An alternative strategy is to reconsider the optimization of the meta-atom. A slight increase in the dielectric spacer could result in a minor reduction in beam deflection efficiency, promoting a more noticeable exposure of the enhanced field into free space and potentially amplifying sensitivity. Another plausible avenue involves incorporating alternative multipolar resonances characterized by higher field confinement, such as toroidal modes and non-radiating anapole modes [49]–[51]. It is noteworthy that the sensing mechanism proposed can also be implemented using all-dielectric metasurfaces. The comparatively lower optical losses in high-index metasurfaces render them more suitable for optical applications in transmission, such as bio-sensing and bio-imaging [52], [53]. Nevertheless, the relatively challenging fabrication process of all-dielectric metasurfaces presents difficulties in sample preparation. Consequently, researchers often choose to employ plasmonic metasurfaces for optical applications in reflection, while reserving dielectric metasurfaces for transmission applications. In addition, we would like to point out that detecting refractive index changes in solutions with absorption remains achievable using the proposed PGMS platform. This is contingent upon the measurability of the intensities of both the specular and deflected beams through the optical setup. As shown in Figure S7, the cross-polarized conversion efficiency undergoes a noticeable decline with an increasing extinction coefficient (*k*). Conversely, the co-polarized intensity maintains consistency across different extinction coefficients. Despite a decrease in the intensity ratio between the LCP-to-RCP efficiency (deflected beams) and LCP-to-LCP reflection (specular beams) due to heightened absorption in the detected material, which reduces the sensitivity of the metasurface, the PGMS still facilitates refractive index detection for absorbing materials owing to the discernible intensities of both specular and deflected beams. These findings accentuate the PGMS’s potential for diverse applications in broadband sensing, such as biomedical diagnostics and environmental monitoring, within the visible spectrum. In addition to its application for refractive index sensing, the proposed PGMS platform is proficient in detecting chiral molecules. Leveraging the intrinsic property of the PB phase-based gradient metasurface, both LCP and RCP deflected beams can be simultaneously obtained when the incident light is linearly polarized. This capability allows the PGMS to detect circular dichroism in real time, revealing the versatility of the PGMS for various sensing applications. Beyond overcoming the limitations of traditional optical sensors, the developed PGMS charts a path toward advanced and highly sensitive optical sensing technologies in the foreseeable future.

## 4 Methods

### 4.1 Simulation

All simulation results were conducted using the commercial software CST Microwave Studio. For the calculation of the single meta-atom design, a unit cell boundary condition was applied along the *x*-direction and *y*-direction to simulate reflection and phase shift in an array structure. Similarly, beam deflection was simulated using the unit cell boundary condition along the *x*-direction and *y*-direction. The refractive indices of SiO_2_ and PMMA were determined through ellipsometry measurements. The permittivity of Al in the visible regime is characterized by the Drude model.

### 4.2 Fabrication

To begin, a 180-nm-thick layer of photoresist (PMMA A4) was spin-coated onto a pre-prepared silicon substrate, which was then baked on a hot plate at 180 °C for 3 min. Next, we employed electron beam lithography (Elionix ELS-7500EX) with an acceleration voltage of 50 keV and a beam current of 50 nano-amperes to define the nanopatterns on the photoresist. This was followed by a development process using the PMMA developer (MIBK:IPA = 1:3) for 2 min. Subsequently, a 50-nm-thick layer of Al was deposited via thermal evaporation at a rate of 1.0 Å/s. After a 24-h acetone treatment in the lift-off process, the meta-atoms were determined.

## Supplementary information

See Supplementary Material for the supporting content.

## Supplementary Material

Supplementary Material Details
