# Supplementary material for: Exploring plasmonic gradient metasurfaces for enhanced optical sensing in the visible spectrum
Source: Nanophotonics. 2024 Jan 15;13(7):1099–108. doi: 10.1515/nanoph-2023-0809 (PMC11502102; doi:10.1515/nanoph-2023-0809)
Supplement: Supplementary file 1 — Supplementary Material Details [file j_nanoph-2023-0809_suppl_001.pdf]

Supporting Information for

Exploring Plasmonic Gradient Metasurfaces for Enhanced Optical Sensing in the Visible Spectrum

Shih-Hsiu Huang[1] and Pin Chieh Wu[1,2,3,]*

[1] Department of Photonics, National Cheng Kung University, Tainan 70101, Taiwan

[2] Center for Quantum Frontiers of Research & Technology (QFort), National Cheng Kung University, Tainan, 70101, Taiwan

[3] Meta-nanoPhotonics Center, National Cheng Kung University, Tainan, 70101, Taiwan

*E-mail: pcwu@gs.ncku.edu.tw

1. The physical mechanism and operation of Pancharatnam-Berry (PB) phase

The relation between the electric field of the incidence $\boldsymbol{E_i}$ and reflection $\boldsymbol{E_r}$ can be expressed using the Jones matrix in Cartesian basis:

$$\begin{bmatrix} E_{rx} \\ E_{ry} \end{bmatrix} = J \begin{bmatrix} E_{ix} \\ E_{iy} \end{bmatrix} \quad where\ J = \begin{bmatrix} J_{xx} & J_{xy} \\ J_{yx} & J_{yy} \end{bmatrix} \tag{S1}$$

Following a rotation by an angle θ, the updated Jones matrix can be described as:

$$\begin{bmatrix} E_{rx} \\ E_{ry} \end{bmatrix} = R^{-1} J\, R \begin{bmatrix} E_{ix} \\ E_{iy} \end{bmatrix} = J_R \begin{bmatrix} E_{ix} \\ E_{iy} \end{bmatrix} \quad where\ R = \begin{bmatrix} cos\theta & -sin\theta \\ sin\theta & cos\theta \end{bmatrix} \tag{S2}$$

Then, the connection between $\boldsymbol{E_i}$ and $\boldsymbol{E_r}$ can be derived in the circular basis:

$$\begin{bmatrix} E_{rLCP} \\ E_{rRCP} \end{bmatrix} = \Lambda^{-1} J_R \Lambda \begin{bmatrix} E_{iLCP} \\ E_{iRCP} \end{bmatrix} \quad where\ \Lambda = \frac{1}{\sqrt{2}} \begin{bmatrix} 1 & 1 \\ i & -i \end{bmatrix} \tag{S3}$$

To simplify, it can be also described as

$$\begin{bmatrix} E_{rLCP} \\ E_{rRCP} \end{bmatrix} = \begin{bmatrix} J_{LL} & J_{LR} e^{i2\theta} \\ J_{RL} e^{-i2\theta} & J_{RR} \end{bmatrix} \begin{bmatrix} E_{iLCP} \\ E_{iRCP} \end{bmatrix} \tag{S4}$$

where J_{LL}, J_{LR}, J_{RL} and J_{RR} represent the conversion coefficient.

2. Deflected angle of a plasmonic gradient metasurface embedded in different materials

The angle of the deflected beam θ_r is determined by the working wavelength and the phase gradient $\frac{d\phi}{dx}$ at interface II (see **Figure 2A** in the main article). Under a normal illumination, the deflected beam θ_r follows the general Snell's law:

$$\sin\theta_r = \frac{\lambda_0}{2\pi n_d}\frac{d\phi}{dx} \tag{S5}$$

where λ_0 is the wavelength in free space and n_d is the refractive index of the analyte layer. Then, the deflected beam passes through the analyte layer to interface I, and the final deflected angle θ_A follows the Snell's law:

$$n_{air}\sin\theta_A = n_d\sin\theta_r \tag{S6}$$

According to Eqns. (S1) and (S2), the refracted angle θ_A can be calculated and described as:

$$\theta_A = \sin^{-1}\left(\frac{\lambda_0}{2\pi}\frac{d\phi}{dx}\right) \tag{S7}$$

Thus, the final deflected angle θ_A is independent with the refractive index n_d.

3. Optical setup of specular reflection and deflection for refractive index sensing

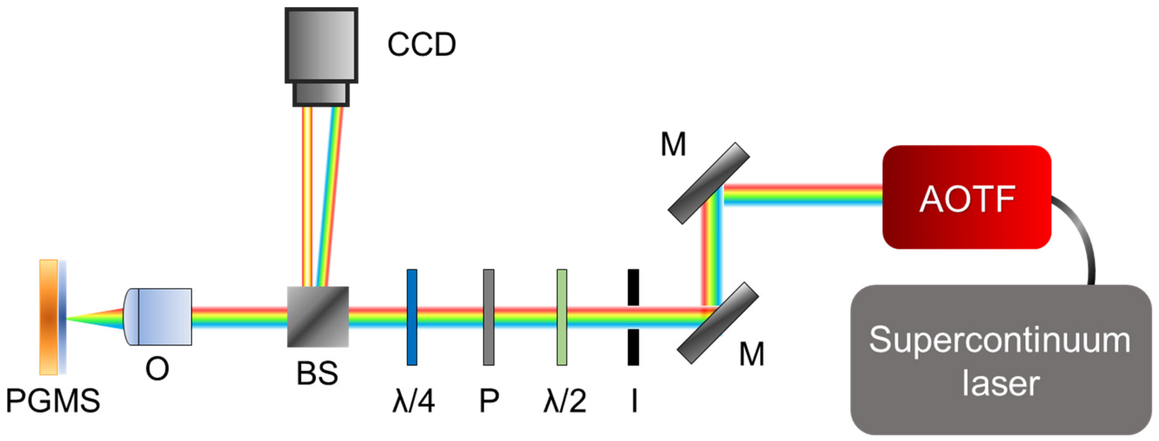

Figure S1. A supercontinuum laser (NKT Photonics FIU-15) combined with an acousto-optic tunable filter (AOTF, SuperK SELECT) is utilized to select the wavelength in the visible. A half-wave plate (Thorlabs AHWP05M-600), a linear polarizer (Thorlabs LPVISE100-A), and a quarter-wave plate (Thorlabs AQWP05M-600) are inserted to determine the polarization state of incident light. M: mirror; I: iris; $\lambda/2$: half-wave plate; P: linear polarizer; $\lambda/4$: quarter-wave plate; BS: beam splitter (Thorlabs CCM5-BS016); O: objective (Mitutoyo 10× magnification with 0.28 numerical aperture).

4. Simulated and measured cross-polarized reflection of the PGMS covered by SiO_2

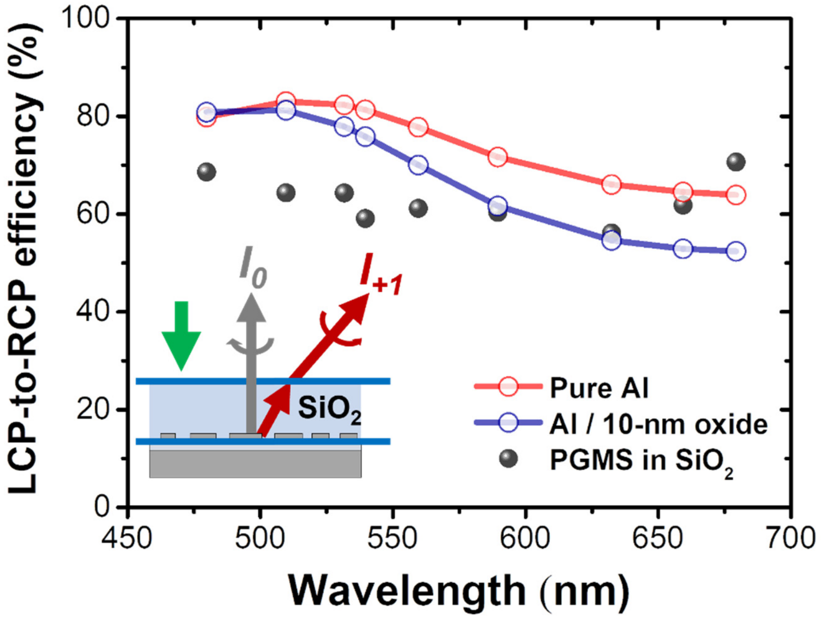

Figure S2. Simulated (red and blue dots) and measured (black circles) cross-polarized conversion efficiency of the deflected beam as the PGMS embedded in SiO_2.

5. Ellipsometry measurement

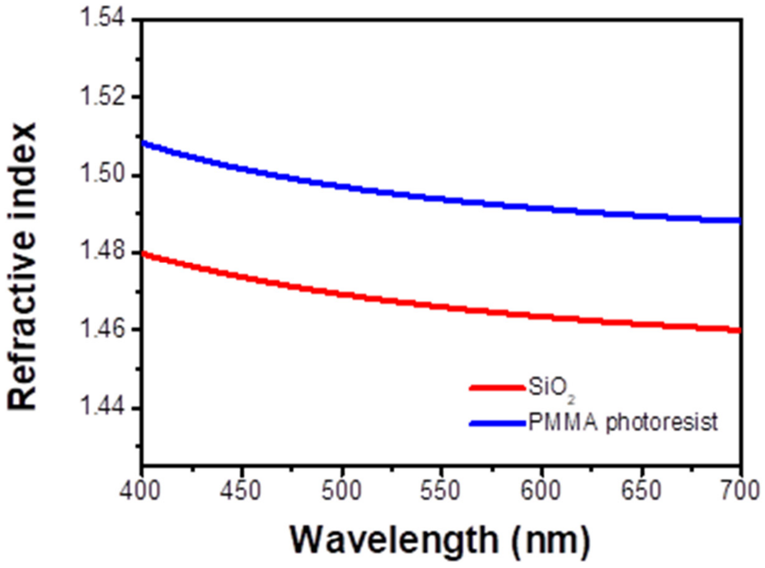

Figure S3. Measured optical refractive indices for SiO_2 (red solid curve) and PMMA photoresist (blue solid curve).

6. Captured CCD images for refractive index sensing

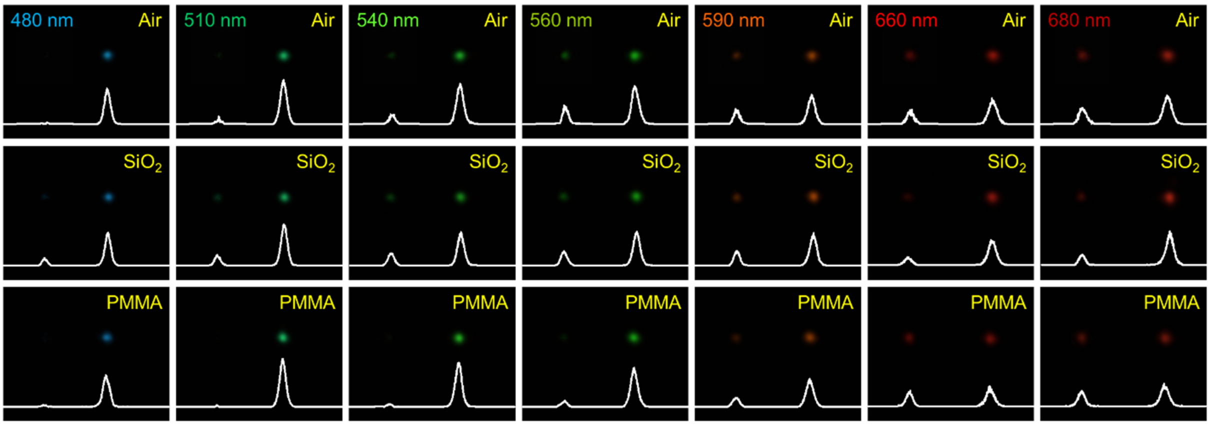

Figure S4. The captured scattered images using the PGMS covered by air, PMMA photoresist, and SiO_2. The working wavelengths are 480, 510, 540, 560, 590, 660, and 680 nm. For each wavelength, both specular and deflected beams were captured at a fixed position.

7. Fitting results of normalized intensity ratio I_{nor}^{*}

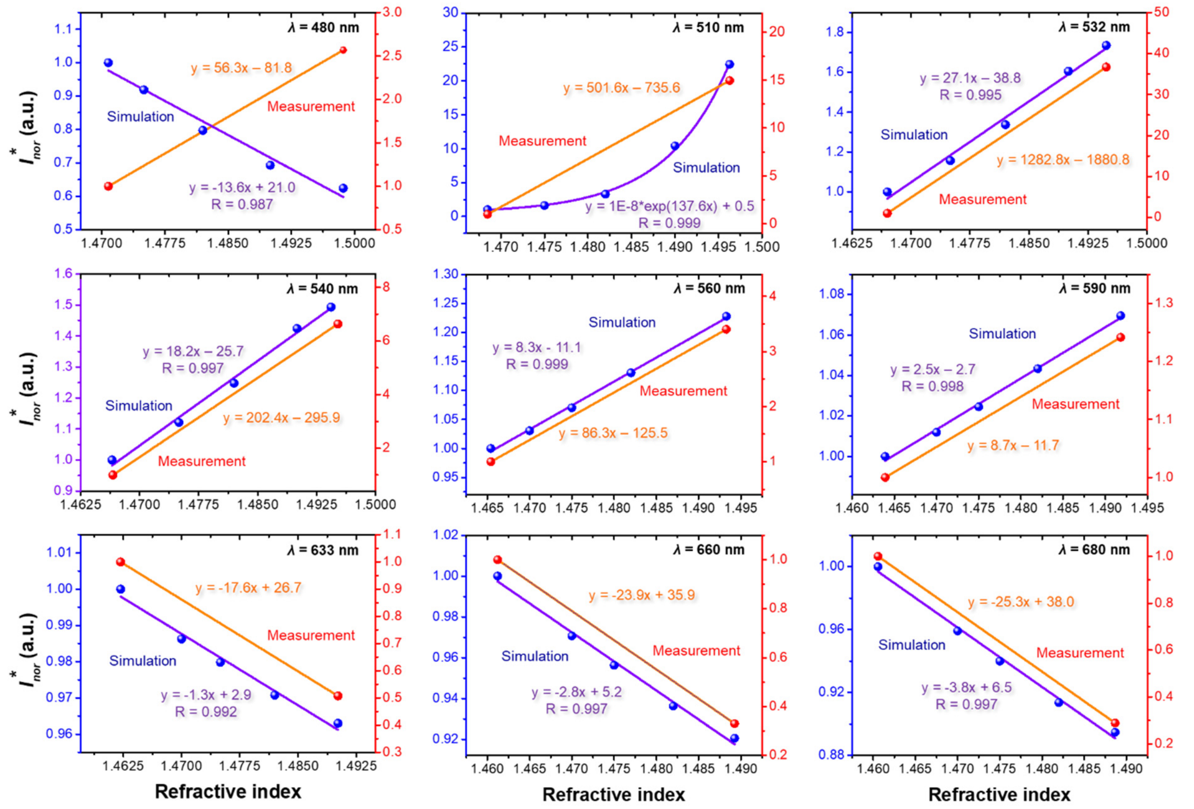

Figure S5. The simulated and measured normalized intensity ratio I_{nor}^{*} across the refractive index range between SiO_2 and PMMA.

8. Simulated field distributions at different wavelengths

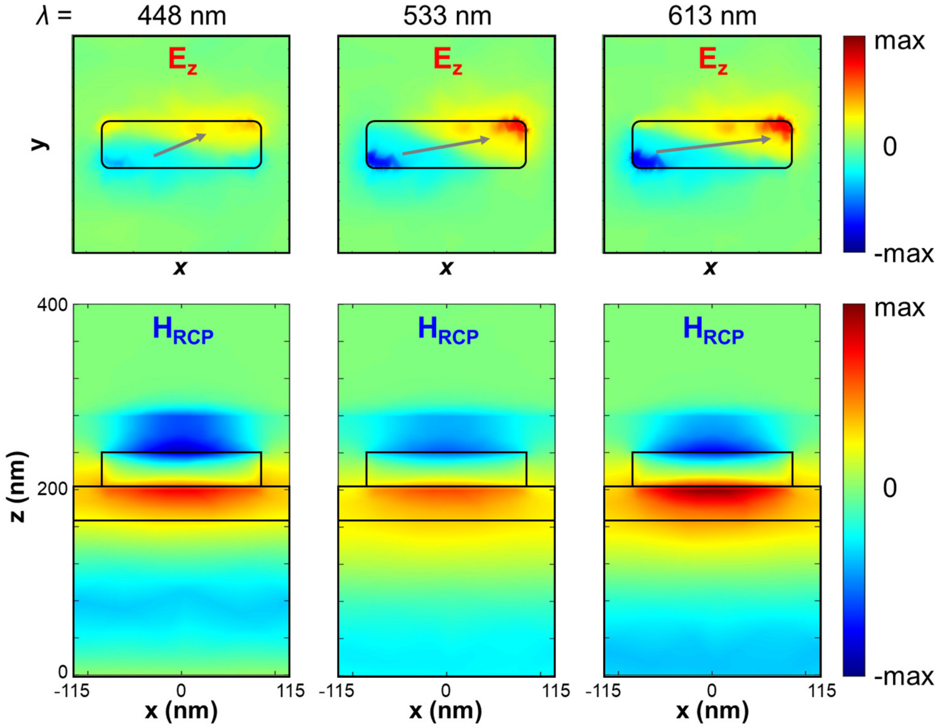

Figure S6. The simulated field distributions are presented at three peak wavelengths, with the corresponding spectrum available in Fig. 1c in the main article. The refractive index of the surrounding environment is set to 1.33. The gray arrows in the top panels indicate the induced effective electric dipole.

9. Co-polarized and cross-polarized spectra of the Al meta-atom

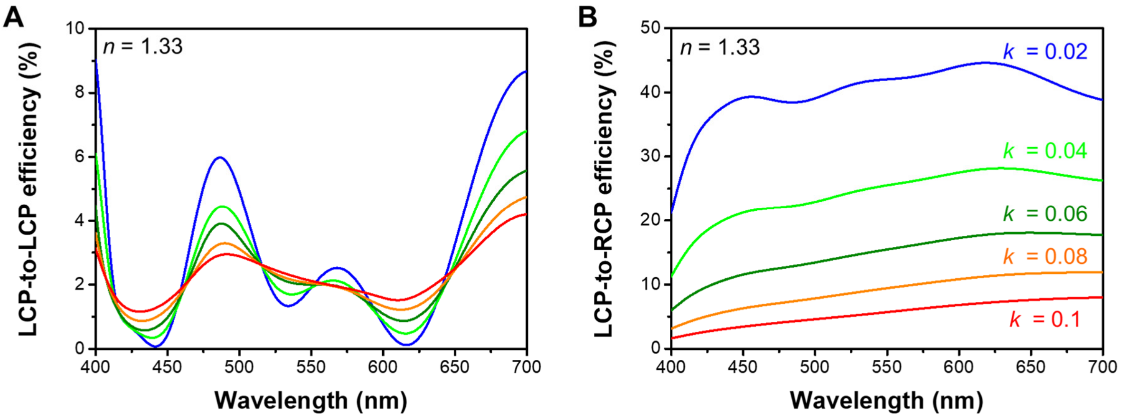

Figure S7. The simulated (A) co-polarized and (B) cross-polarized reflection spectra of the Al meta-atom embedded in a medium with a fixed refractive index of 1.33 and varying extinction coefficients (*k*).

10. Experimentally captured CCD images of incident light beams

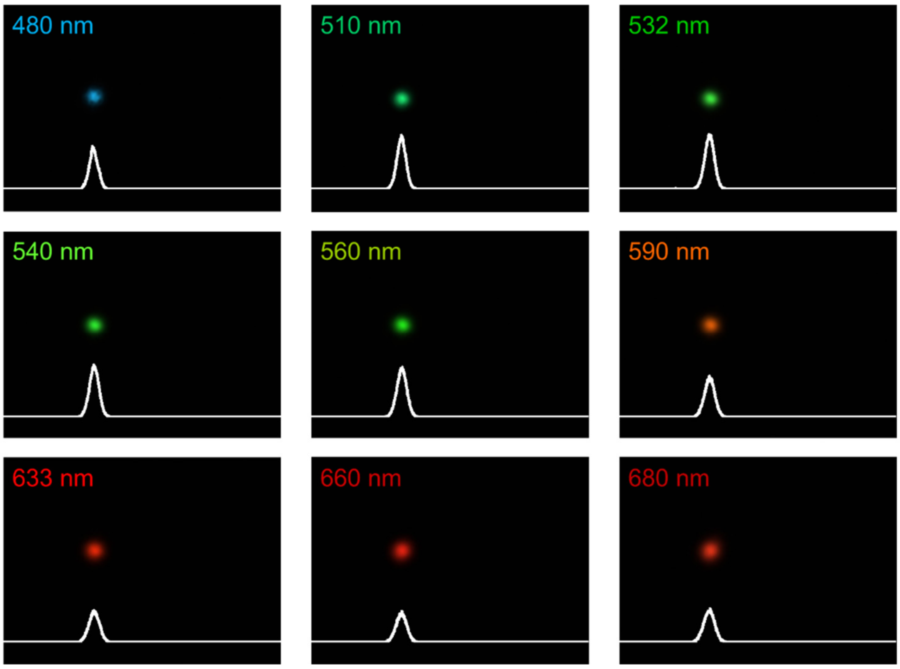

Figure S8. The captured images of incident beams when using a silver mirror as the sample. The working wavelengths are 480, 510,532, 540, 560, 590,633, 660, and 680 nm.
